# First results from five multidisciplinary diagnostic centre (MDC) projects for non-specific but concerning symptoms, possibly indicative of cancer

**DOI:** 10.1038/s41416-020-0947-y

**Published:** 2020-07-06

**Authors:** D. Chapman, V. Poirier, D. Vulkan, K. Fitzgerald, G. Rubin, W. Hamilton, S. W. Duffy, on behalf of the ACE MDC projects, Alan Hart Thomas, Dawn Gulliford, Helena Rolfe, Matthias Hohmann, Chris Repperday, Susan Sykes, Sarah Taylor, Angie Craig, James Dawson, Sarah Forbes, Helen Ryan, Rob Turner, Mush Ahmad, Donna Chung, David Graham, Andrew Millar, Sara Taiyari, Claire Friedemann Smith, Fergus Gleeson, Shelley Hayles, Zoe Kaveney, Brian Nicholson

**Affiliations:** 1 Cancer Research UK, London, UK; 2 Centre for Cancer Prevention, Wolfson Institute of Preventative Medicine, Queen Mary University of London, London, UK; 3 Institute of Population Health Sciences, Newcastle University, Newcastle upon Tyne, UK; 4 University of Exeter Medical School (Primary Care), Devon, UK

**Keywords:** Cancer, Health services

## Abstract

**Background:**

Patients with non-specific symptoms often experience longer times to diagnosis and poorer clinical outcomes than those with site-specific symptoms. This paper reports initial results from five multidisciplinary diagnostic centre (MDC) projects in England, piloting rapid referral for patients with non-specific symptoms.

**Methods:**

The evaluation covered MDC activity from 1st December 2016 to 31st July 2018, with projects using a common dataset. Logistical regression analyses were conducted, with a diagnosis of any cancer as the dependent variable. Exploratory analysis was conducted on presenting symptoms and diagnoses of cancer, and on comparisons within these groupings.

**Results:**

In total, 2961 patients were referred into the MDCs and 241 cancers were diagnosed. The pathway detected cancers across a broad range of tumour sites, including several rare and less common cancers. An association between patient age and cancer was identified (*p* < 0.001). GP ‘clinical suspicion’ was identified as a strong predictor of cancer (*p* = 0.006), with a reduced association with cancer observed in patients with higher numbers of GP consultation before referral (*p* = 0.008).

**Conclusions:**

The MDC model diagnoses cancer in patients with non-specific symptoms, with a conversion rate of 8%, demonstrating the diagnostic potential of a non-site-specific symptomatic referral pathway.

## Background

Patients presenting with non-specific but concerning symptoms (hereafter ‘non-specific symptoms’) account for a significant proportion of cancer diagnoses in England.^1^ Some of these symptoms, such as unexplained weight loss, non-specific abdominal pain, fatigue and nausea/vomiting, have a low predictive value for individual cancers.^2–4^ However, whilst the risk of specific cancers may be low, the overall risk of harbouring cancer of any type is higher,^2,5^ suggesting that swift investigation for patients with non-specific symptoms is merited. In the absence of an established pathway to ensure a timely and planned referral, these patients have often experienced longer times to diagnosis than those presenting with recognised high-risk symptoms indicative of specific cancers.^4,6,7^

As non-specific symptoms can be caused by a range of conditions, of which cancer is only one, identifying the appropriate diagnostic test and referral route can be challenging. Consequently, patients with non-specific symptoms more frequently have multiple GP consultations before referral,^6–8^ potentially contributing to longer intervals from presentation to diagnosis of cancer.^6,7^ Patients in this cohort are also associated with higher rates of cancer diagnosis via emergency presentation^6,9^ and of late-stage cancer diagnoses,^6^ both of which contribute to poorer clinical outcomes^10–12^ and poorer patient experience of care.^13^

The multidisciplinary diagnostic centre (MDC) concept aims to improve outcomes for patients presenting with non-specific symptoms by providing rapid access to a range of diagnostic tests within a single diagnostic pathway, with a number of specialists working together to speed up diagnosis for the patient.^11^ The MDC concept was first trialled nationally in Denmark in 2012 as part of its three-legged cancer strategy,^14–16^ with Diagnostic Centres established alongside arrangements for the investigation of low-risk but not no-risk symptoms (Yes/No Clinic), and an urgent referral pathway for specific alarm symptoms. The Danish pathway comprises a filter function conducted by the GP, which includes a range of pre-specified diagnostic tests, followed by referral into the Diagnostic Centre itself if serious symptoms persist without a diagnosis being reached.^14^

The Accelerate Coordinate Evaluate (ACE) Programme^17^ of interventions is aimed at improving the pathway to cancer diagnosis, and thereby improving cancer outcomes, through the provision of evidence-based information and support. Wave 2 of the programme involved the establishment of a cluster of five projects in England to examine and evaluate the concept of MDC-based pathways for patients presenting with non-specific symptoms.

In this study, we evaluate the early outcomes for the ACE Programme’s pilot MDC sites, in respect to patient characteristics, cancer diagnoses and stage at diagnosis, and associations between patient factors and diagnosis of cancer.

## Methods

### Project structure

ACE MDC Programme activity was based within five projects in England, with ten operational MDC pilot sites (Airedale, Greater Manchester (×2), Leeds, London (×5) and Oxford). All projects focused on the development of a pilot pathway for patients with non-specific symptoms and, whilst projects configured their approaches to reflect local healthcare systems and clinical priorities, a core set of distinguishing features were evident across all pilot sites.^18^

As the programme was structured around a service evaluation of pilot pathways, a level of heterogeneity was introduced amongst the projects to test differing MDC approaches and to assess pathway adaptability. However, regarding the clinical interpretation of the patient’s presenting symptoms, all five projects adhered to two fundamental principles for referral:

1. That the patient must be considered as being of clinical concern, with non-specific symptoms potentially indicative of cancer (or other serious disease) and
2. That their presenting symptoms are not sufficiently clear to indicate an appropriate tumour-specific urgent referral pathway.

As the MDC model focused on the diagnosis of any cancer presenting with non-specific symptoms, and not being limited to new cancer cases only, the presence of a previous cancer was permitted, provided that the patient met the pathway’s two governing principles for referral. Despite being considered a site-specific symptom, painless jaundice was included as a referral criterion in London MDC to reflect locally determined clinical priorities.

Table 1 provides details of the date at which the intervention was implemented, and the referral routes and the criteria for each MDC project.

**Table 1:** Initial MDC arrangements by individual project.

*Airedale*	
Launch date	17th January 17
Referral criteria	Persistent unexplained abdominal pain, persistent unexplained weight loss, non-specific but concerning symptoms with a high risk of cancer, GP clinical suspicion and too unwell for 2-Week Wait referral
Referral route	GP, A&E and Secondary Care Clinic
*Greater Manchester*	
Launch date	3rd Mar 17 (Royal Oldham Hospital), 13th December 16 (Wythenshawe Hospital)
Referral criteria	Non-specific abdominal pain, unexplained weight loss, severe unexplained fatigue, nausea/appetite loss, lymphadenopathy, hepatomegaly, splenomegaly, bloating, GP clinical suspicion and non-iron- deficiency anaemia
Referral route	GP
*Leeds*	
Launch date	31st January 17
Referral criteria	Appetite loss + nausea (unexplained, 40 and over), weight loss (unexplained, 40 and over), abdominal pain without rectal bleeding or weight loss (<3-month duration or recent change in character/severity, 50 and over), anaemia (non-iron deficiency, without evidence of bleeding, 50 years and over), hypercalcaemia (unexplained and persisting <12 months), thrombocythemia (unexplained and persisting <12 months and GP clinical suspicion and general condition (“poor” general condition)
Referral route	GP, Acute Medicine
*London*	
Launch date	1st May 17 (North Middlesex), 1st April 17 (UCLH), 1st December 16 (Queens—BHRUT), 1st January 18 (Royal Free Hospital) and 15th September 2017 (Southend)
Referral criteria	Broad range of abdominal symptoms with no clear referral pathway and where patients cannot wait for routine referral, including new unexplained abdominal pain, unexplained weight loss, persistent nausea/appetite loss, GP clinical suspicion and painless jaundice
Referral route	GP
*Oxford*	
Launch date	15th March 17
Referral criteria	Severe unexplained fatigue, unexplained weight loss, persistent nausea or appetite loss, new atypical pain, unexplained laboratory findings, no organ-specific symptoms, no symptoms fulfilling referral via the standard 2-week wait pathway, over 40 years old and GP clinical suspicion (“gut feeling”)
Referral route	GP

### Research methods

A programme dataset was agreed across the projects to ensure a robust evaluation. It included data items based on the English cancer outcomes and services dataset,^19^ and additional project- specific items focusing on secondary care presentation, diagnostic process of cancers and other diseases. Data items were collected locally from a combination of primary care referral forms and secondary care data systems.

Data management arrangements varied between MDC projects; a combination of local healthcare IT systems and stand-alone systems were used, which impacted upon the availability of certain data, for example, missing performance status and comorbidity data items. The data items were captured in near real time where possible either by automated data extraction or entry by clinical staff. Some additional data required manual entry at a later date.

All projects had a nominated data lead, and project data items were submitted to programme evaluators on a quarterly basis, where some recoding rules were applied to align the data for analysis.

A list of thirteen symptoms (plus ‘other’) was included in the agreed dataset. In subsequent analyses, these thirteen symptoms are referred to together as ‘selected symptoms’. The range of selected symptoms was developed with clinical guidance and chosen to describe a general pattern of seriously unwell patients, whose presentation is suggestive of cancer, but does not indicate a specific diagnostic approach. In some instances, this includes conditions and signs that are not strictly symptoms. In addition to the thirteen ‘selected symptoms’, other symptoms were recorded as part of open-ended data recording; these have been classified as ‘non-selected’ symptoms.

Patients presented with non-specific symptoms in either isolation or in varying combinations. Clinical judgement formed part of the decision to refer, and all relevant factors were considered as part of this process and captured on referral templates where relevant. Eligible referral criteria were included on MDC referral templates to aid consistency and quality, and to clearly indicate patient symptoms as appropriate.

As indicated in Table 1, individual MDC hospital sites became operational at different times and began contributing data at different points during the evaluation period, from December 2016 to January 2018, with this evaluation covering MDC activity up to 31st July 2018.

As projects have uniformly applied their selected referral criteria, filter tests and diagnostic approaches to their own pilot sites, data in this study are aggregated to a project and programme level to provide greater scope for analysis. Although measures were taken centrally to support data consistency and completeness, a certain degree of variation in data interpretation and formatting was created at MDC level. Additional variations in the data completeness across the pilot sites were observed, often due to manpower issues. Certain data items have approximately one-third of data classified as ‘unknown’. Performance status^20^ was not recorded for 897 patients (30% of the total), and comorbidity^21^ information was absent for 729 (25%). Performance status and comorbidity, both of which had relevant data recorded for four MDC projects only, were retained for the purpose of describing the characteristics of the overall cohort.

The number of primary care consultations was missing for 1351 patients (46%), and symptom duration for 1012 (34%). In each case, the extent of missing data varied by project site. Sensitivity analyses have been conducted on primary care consultation history and symptom duration to assess the likely impact of missing data, as these data items have been included in logistic regression analyses. Missing data are noted as unknown in the tables, and records with missing data have been excluded from all analyses relating to those fields (i.e. blanks have been omitted from analyses).

### Statistical analysis

Patients were deemed to have a cancer if their diagnostic outcome was recorded as either “New cancer” or “Recurrence”.

A series of single-variable logistic regressions were carried out, with diagnosis of any cancer as the dependent variable. Independent variables considered were gender, age, number of primary care consultations prior to referral (up to three, or three or more), duration of symptoms (five categories), the presence of each of the thirteen selected symptoms, including one general symptom (the classification of ‘pain’ as a collective grouping of all pain-associated symptoms, 85% of cases being abdominal pain) and the number of selected symptoms reported.

To assess the likely impact of missing data relating to previous primary care consultations and reported duration of symptoms, two approaches were undertaken. The first approach was to omit those sites where, for each variable considered, no data were recorded for 20% or more of patients. The second approach was to replace all missing values with, in turn, the highest or lowest possible values of the variable, therefore creating a ‘best case’ and ‘worst case’ scenario for each variable. The results carried out using these approaches did not provide strong evidence to reconsider the conclusions reached. The results are presented in Supplementary Information A.

Further logistic regression was carried out, taking multiple independent variables into account at the same time. Statistically significant (at the 0.05 level) factors identified in the single-variable analyses (gender, age, fewer than 3 previous primary care consultations, total number of selected symptoms, GP ‘clinical suspicion’, nausea/appetite loss, fatigue and anaemia) were included in multiple regression to assess possible confounding effects. Multivariable analyses are presented as ‘adjusted’ rates alongside the relevant ‘unadjusted’ single-variable rates.

## Results

There were 2961 patients referred to the pilot MDCs. Table 2 shows the demographic characteristics, comorbidity score and primary care consultation history of the patients referred. Patients’ ages ranged from 17 to 97 years, with an average of 66.7 (SD 14.9). Of the patients, 44% were male, 40% had some degree of physical impairment (based on recorded performance status of 2064 completed patient records) and 27% had moderate or severe comorbidities (based on 2232 completed patient records). Of those with known primary care consultation history, 28% had 3 or more previous consultations.

**Table 2:** Characteristics of the patients referred to the MDC.

Total number of patients	2961	
*Sex*	*N*	*%*
Male	1304	44.2
Female	1646	55.8
Unknown	11	N/A
*Age group*		
Under 30	52	1.8
30–39	109	3.7
40–49	231	7.8
50–59	480	16.2
60–69	649	21.9
70–79	814	27.5
80–89	567	19.2
90–99	59	2
Mean (SD)	66.7 (14.9)	
Median	69	
*Performance status*		
0	1239	60.0
1	462	22.4
2	232	11.2
3	113	5.5
4	18	0.9
Unknown	897	N/A
*Comorbidity score*		
0	699	31.3
1	938	42.0
2 or 2/3	406	18.2
3	189	8.5
Unknown	729	N/A
*Primary care consultations*		
None	114	7.1
1	539	33.5
2	501	31.1
3	221	13.7
4	91	5.7
5	50	3.1
More than 5	94	5.8
Unknown	1 351	N/A

Table 3 shows the frequency of selected symptoms of referred patients. The most common symptom was weight loss (66% of those for whom symptoms were recorded) followed by GP ‘clinical suspicion’ (36%) based on the GP’s clinical judgement regarding overall suspicion of malignancy^2^ and pain (32%). In total, 61% of patients presented with more than one of the thirteen selected symptoms (decreasing to 57% when including non-selected symptoms, but excluding GP ‘clinical suspicion’ and family concern). Of those with symptom duration data, 55% reported a symptom duration of 3 months or more.

**Table 3:** Nature, number and duration of symptoms in patients referred to the MDC.

Reported symptoms	As only selected symptom		With other symptoms		All occurrences	
	*N*	%	*N*	%	*N*	%
Weight loss	579	20.3	1,295	45.4	1874	65.7
GP clinical suspicion	124	4.3	904	31.7	1028	36.1
Pain	248	8.7	678	23.8	926	32.5
Nausea/appetite loss	26	0.9	817	28.7	843	29.6
Fatigue	18	0.6	528	18.5	546	19.2
Anaemia	40	1.4	256	9	296	10.4
Change in bowel habit	9	0.3	119	4.2	128	4.5
General condition	6	0.2	97	3.4	103	3.6
Respiratory problem	5	0.2	67	2.4	72	2.5
Thrombocytosis	4	0.1	19	0.7	23	0.8
Jaundice	9	0.3	10	0.4	19	0.7
Hypercalcaemia	0	0	8	0.3	8	0.3
New-onset diabetes	0	0	3	0.1	3	0.1
Other symptom(s) (only)	43	1.5	N/A	N/A	43	1.5
Unknown					110	N/A
*Number of selected symptoms reported*					*N*	%
None					43	1.5
1					1068	37.5
2					879	30.8
3					515	18.1
4					244	8.6
5					90	3.2
6					12	0.4
Overall number of symptoms per case**						
1 or fewer					1234	43.3
2 or more					1617	56.7
*Reported symptom duration*						
Less than 1 week					25	1.3
1–4 weeks					348	17.9
5–12 weeks					507	26.0
3–6 months					573	29.4
Over 6 months					496	25.5
Unknown					1012	N/A

**Including non-selected symptoms, excluding GP clinical suspicion and patient/family concern.

Of the patients referred, 240 (8%) were diagnosed with cancer, 239 with a single cancer and one patient diagnosed with two cancers (one urological and one lower GI). In the 16 cases where cancer was classified as ‘recurrence’, all patients presented without any recognised alarm symptoms. In addition to diagnoses of cancer, over 50% of cases within the MDC were diagnosed with non-cancer conditions; of those with a given diagnosis, 40% were related to diseases of the digestive system.

Table 4 shows the sites of the cancers diagnosed. The commonest were upper GI (including 25 cases of pancreatic cancer) and lung, with 53 and 52 cancers diagnosed, respectively.

**Table 4:** Anatomical sites of the 240 patients diagnosed with cancer.

Cancer site	Number	%
Upper GI	53	22
Lung	52	22
Haematological	31	13
Lower GI	30	13
Urological	30	13
Breast	15	6
Sarcoma	6	3
Lower GI and urological	1	0
Other	20	8
Unknown	2	1

Table 5 shows the results of logistic regression for associations of selected factors with a diagnosis of any cancer. When considered in isolation, several factors displayed strong associations (*p* < 0.05) with a diagnosis of cancer. However, when accounting for all significant individual variables at the same time, strong associations were only identified between a cancer diagnosis and increasing age (*p* < 0.001), and with GP ‘clinical suspicion’ (*p* = 0.006) as a predictor of cancer. In addition, an association was maintained between patients with more than three GP consultations prior to referral (*p* = 0.008) and a reduced likelihood of a cancer diagnosis.

**Table 5:** Logistic regression results for associations of selected factors with diagnosis of cancer.

Category—factor	Cancer *N* (%)	No cancer *N* (%)	Single variable ‘unadjusted'		Multivariable ‘adjusted’**	
			Odds ratio (95% CI)	Significance	Odds ratio (95% CI)	Significance
*Sex*						
Male	122 (9.4)	1 182 (90.6)	1.00	**0.027**	1.00	*0.172*
Female	117 (7.1)	1 529 (92.9)	**0.74 (0.57–0.97)**		*0.77 (0.53–1.12)*	
*Age*						
Per year of age	N/A		**1.04 (1.03–1.06)**	<0.001*	**1.05 (1.03–1.07)**	<0.001*
*Previous 1° care consultations*						
<= 3	122 (8.9)	1 253 (91.1)	1.00	**0.002**	1.00	**0.008**
> 3	6 (2.6)	229 (97.4)	**0.27 (0.12–0.62)**		**0.32 (0.14–0.75)**	
*Duration of symptoms*						
<1 week	2 (8.0)	23 (92.0)	1.00	**0.038**	**–**	**–**
1–4 weeks	43 (12.4)	305 (87.6)	*1.62 (0.37–7.12)*		**–**	
5–12 weeks	44 (8.7)	463 (91.3)	*1.09 (0.25–4.79)*		**–**	
3–6 months	49 (8.6)	524 (91.4)	*1.08 (0.25–4.70)*		**–**	
>6 months	30 (6.0)	466 (94.0)	*0.74 (0.17–3.29)*		**–**	
*Symptoms (selected)*						
Weight loss	N: 76 (7.8)	901 (92.2)	*1.05 (0.79–1.39)*	*0.76*	*–*	*–*
	Y: 152 (8.1)	1 722 (91.9)				
GP clinical suspicion	N: 113 (6.2)	1 710 (93.8)	**1.91 (1.45–2.50)**	**<0.001**	**1.88 (1.20–2.94)**	**0.006**
	Y: 115 (11.2)	913 (88.8)				
Pain	N: 154 (8.0)	1 771 (92.0)	*1.0 (0.75–1.33)*	*0.99*	*–*	*–*
	Y: 74 (8.0)	852 (92)				
Nausea/appetite loss	N: 144 (7.2)	1 864 (92.8)	**1.43 (1.08–1.90)**	**0.012**	*1.18 (0.72–1.95)*	*0.51*
	Y: 84 (10.0)	759 (90.0)				
Fatigue	N: 166 (7.2)	2 139 (92.8)	**1.65 (1.21–2.25)**	**0.001**	*1.29 (0.75–2.20)*	*0.36*
	Y: 62 (11.4)	484 (88.6)				
Anaemia	N: 186 (7.3)	2 369 (92.7)	**2.11 (1.47–3.02)**	**<0.001**	*1.16 (0.63–2.13)*	*0.64*
	Y: 42 (14.2)	254 (85.8)				
Change in bowel habit	N: 219 (8.0)	2 504 (92.0)	*0.86 (0.43–1.72)*	*0.68*	*–*	*–*
	Y: 9 (7.0)	119 (93.0)				
General condition	N: 222 (8.1)	2526 (91.9)	*0.70 (0.31–1.62)*	*0.41*	*–*	*–*
	Y: 6 (5.8)	97 (94.2)				
Respiratory problem	N: 220 (7.9)	2 559 (92.1)	*1.45 (0.69–3.07)*	*0.33*	*–*	*–*
	Y: 8 (11.1)	64 (88.9)				
Thrombocytosis	N: 226 (8.0)	2602 (92.0)	*1.10 (0.25–4.71)*	*0.9*	*–*	*–*
	Y: 2 (8.7)	21 (91.3)				
Jaundice	N: 226 (8.0)	2606 (92.0)	*1.36 (0.31–5.91)*	*0.69*	*–*	*–*
	Y: 2 (10.5)	17 (89.5)				
Hypercalcaemia	N: 227 (8.0)	2616 (92.0)	*1.65 (0.20–13.44)*	*0.64*	*–*	*–*
	Y: 1 (12.5)	7 (87.5)				
New onset of diabetes	N: 228 (8.0)	2620 (92.0)	*N/A*	*N/A*	*–*	*–*
	Y: 0 (–)	3 (100.0)				
*Number of selected symptoms*						
Per additional selected symptom	N/A		**1.34 (1.20–1.49)**	<0.001*	*1.09 (0.83–1.43)*	0.53*

*Trend test.

**Multivariable analyses on gender, age, fewer than 3 previous primary care consultations, total number of selected symptoms, GP clinical suspicion, nausea/appetite loss, fatigue and anaemia (pseudo-R2 = 0.0921).

Bold values in the table denote significant results, whereas italic values denote non-significant results.

The remaining factors, when included in multiple regression, no longer recorded a statistically significant association with cancer diagnosis, namely, gender (*p* = 0.172), nausea/appetite loss (*p* = 0.51), fatigue (*p* = 0.36), anaemia (*p* = 0.64) and the number of symptoms reported (*p* = 0.53).

Table 6 shows the stage distribution of cancers diagnosed. For all cancers, the majority were at stage III or IV. There were, however, notable proportions at stage I and II for upper GI, lung and haematological cancers. For the haematological cancers, however, stage was unavailable for more than 50% of the cancers.

**Table 6:** Stage distributions of cancers diagnosed.

Cancer type	Stage 1	Stage 2 (incl. 1/2)	Stage 3	Stage 4 (incl. 3/4)	Known stage	Unknown stage	Total
	*N* (%)	*N* (%)	*N* (%)	*N* (%)			
Upper GI	2 (4.3)	10 (21.3)	8 (17.0)	27 (57.4)	47	6	53
Lung	12 (25.5)	4 (8.5)	6 (12.8)	25 (53.2)	47	5	52
Lower GI	0 (–)	5 (17.2)	10 (34.5)	14 (48.3)	29	2	31
Urological	4 (16.7)	2 (8.3)	4 (16.7)	14 (58.3)	24	7	31
Haematological	3 (20)	2 (13.3)	6 (40)	4 (26.7)	15	16	31
Breast	4 (33.3)	0 (–)	0 (–)	8 (66.7)	12	3	15
Sarcoma	0 (–)	1 (16.7)	4 (66.7)	1 (16.7)	6	0	6
Other type	0 (–)	0 (–)	1 (7.7)	12 (92.3)	13	7	20
Unknown	0	0	0	0	0	2	2
Total	25 (13.0)	24 (12.4)	39 (20.2)	105 (54.4)	193	48	241

## Discussion

The most important aspect of the study is that 8% of referrals resulted in a cancer diagnosis, with 241 cancers diagnosed from a referral cohort of 2961 patients. Thus, this study of activity within the ACE pilot sites has shown that MDC-based pathways diagnose cancer in patients presenting with non-specific symptoms. Although most referrals do not result in a cancer diagnosis for the patient, the reported conversion rate of 8% exceeds the positive predictive value of 3% recommended for urgent definitive investigation,^2^ and remains consistent with national guidance.

The MDCs diagnosed cancers across a broad range of tumour sites, which is to be expected given the purpose of the pathway and the non-specific symptoms of patients. Encouragingly, several of these diagnoses were of rare and less common cancers, reflecting the higher risk of harbouring cancer of any type amongst this patient group.^2,5^

Patient age was found to be the most important factor associated with a cancer diagnosis (*p* < 0.001) and, once age was controlled for, the significance of other factors was reduced. A significant relationship was also detected between a diagnosis of cancer and GP ‘clinical suspicion’ (*p* = 0.006), and is consistent with GP ‘clinical suspicion’ being a powerful predictor of cancer.^22^ The reduced association between a cancer diagnosis and patients with more than three GP consultations prior to referral is also noteworthy, despite partial data completeness being observed at a project level. Within the context of this study, it may be that where they have a strong suspicion of cancer, GPs are more likely to pursue available options such as the MDC to achieve earlier referral for these patients. Consequently, many of the cases of cancer in this study may have been suspected and referred by the GP during the first few consultations. Analyses of a similar patient cohort suggest that, without a rapid referral route such as the MDC, cancer patients with non-specific symptoms often experienced greater numbers of GP consultations before referral than those with site-specific symptoms,^6^ thus highlighting the potential for this new approach.

The study has provided information that may be useful in describing this new referral cohort. Notable proportions of patient referrals in four MDC projects reported poor performance and comorbidity. A strong association between these factors and increasing age obfuscates the nature and significance of these characteristics,^23,24^ but is useful in illustrating the complexity of diagnostic decision-making for this patient group.

Several presenting symptoms recorded increased odds of a cancer diagnosis, most notably fatigue (OR 1.29 (0.75–2.20)), nausea/appetite loss (OR 1.18 (0.72–1.95)) and anaemia (OR 1.16 (0.63–2.13)), whilst the risk of cancer also marginally increased per additional symptom reported (OR 1.09 (0.83–1.43)), although none of these were statistically significant. There was no clear effect of the reported duration of symptoms overall, but data suggested an increased association for a duration of 1–4 weeks, which then declined for longer durations. However, this may be influenced by challenges regarding objective and accurate symptom recall,^3^ and a higher degree of precision in remembering the onset of more recent symptoms.^25^ Collectively, such information may be helpful in describing the type of patient and presenting a profile that may benefit from urgent referral for non-specific symptoms.

There were relatively large proportions of subjects with stage IV cancer. As several of the non-specific symptoms are systemic, they are consistent with metastatic cancer. However, a substantial proportion of early-stage lung, upper GI tract and haematological cancers were diagnosed, indicating that such symptoms may also provide an opportunity for timely diagnosis via a dedicated referral pathway such as the MDC, for these cancers at least. Furthermore, recent research indicates that a notable proportion of some solid tumour cancers presenting with non-specific symptoms were diagnosed at stages 1–3 (e.g. patients presenting with abdominal pain (as single symptom: 67%, with other symptom(s): 62%)), suggesting that early diagnosis for these patients remains a possibility.^26^

It is possible that a number of cancers, including those at early stage, were diagnosed ‘incidentally’, in that the symptom or symptoms leading to referral were not caused by the cancer. However, this does not necessarily mean that the symptoms were not related to the cancer.^27,28^ They may, for example, have been of chronic non-malignant diseases sharing risk factors with the cancer.^29,30^

There are some notable limitations. Firstly, this is a cohort study with no comparison group. This, in addition to the time-limited nature of the evaluation, has limited the ability to make judgements on the relative impact of the pathway being trialled and its long-term effect on patient outcomes. Whilst MDCs have provided an alternative rapid referral pathway for patients with suspected serious illness, further evaluative assessment should consider the balancing of benefit and harm regarding diagnostic investigations amongst this cohort, and for whom a cancer diagnosis occurs for a minority of patients. There was some missing data associated with the evaluation arrangements and, although measures were implemented to promote consistency regarding data collection and reporting, a certain degree of variation was evident due to localised data arrangements, something that warrants detailed consideration regarding the development of national data collection arrangements for similar pathways. Therefore, the study is dependent on the quality and completeness of the data collected. Finally, the study is based on a relatively small sample size that should be considered when assessing the pathway’s key findings.

The ACE Programme has evaluated MDC-based pathways for the diagnosis of cancer amongst patients with non-specific symptoms. Additional analyses on MDC diagnostic testing activity and the results will elaborate on programme heterogeneity and associated learning, and will contribute to existing descriptive materials^18^ from the evaluation. Further analyses are also planned regarding the diagnosis of non-cancer conditions. Programme learning to date has informed the national development and implementation of rapid diagnostic centres (RDC)^31^ in England as part of the NHS Long Term Plan.^32^

It will be necessary to conduct further research on the longer-term impact of MDC pathways on outcomes for patients presenting with non-specific symptoms, and to build on existing work to assess patient experience within the MDC.^33^ The development of a viable comparator dataset will be essential to support future evaluative assessments, including a full evaluation of pathway health economics. These areas fall outside of the specific focus of this paper, but will provide valuable additional and complementary information in support of an emerging evidence base.

## Conclusions

This evaluation of MDC pilots in England has demonstrated the potential of a dedicated referral pathway for patients presenting with non-specific but concerning symptoms, and provides a platform for the further development of the subject’s evidence base. We found a strong association between a cancer diagnosis and patient age, and have identified GP ‘clinical suspicion’ as a strong predictor of cancer within this non-specific symptom cohort. This study has demonstrated that a cancer referral pathway for patients with non-specific but concerning symptoms could be a valuable addition to the referral options for suspected cancer, across a broad range of cancer sites.

## ACE MDC projects

*Airedale MDC pilot*: Dr Alan Hart Thomas, Respiratory Consultant; Dawn Gulliford, Cancer Patient Services manager; Dr Helena Rolfe, Cancer Lead GP; Airedale MDC clinical team. *Greater Manchester MDC pilots*: Dr Matthias Hohmann, Oldham GP Cancer Lead; Chris Repperday, Data Analyst; Susan Sykes, Senior Programme Manager; Dr Sarah Taylor, Greater Manchester GP Cancer Lead; Greater Manchester MDC clinical teams. *Leeds MDC pilot*: Angie Craig, LTHT Assistant Director of Operations and Diagnostic Lead; James Dawson, Assistant Information Manager; Dr Sarah Forbes, GP Cancer Lead; Helen Ryan, Macmillan Leeds Cancer Programme Project Lead (Early Diagnosis); Dr Rob Turner, Consultant Clinical Oncologist. Leeds ACE MDC Clinical Team and Steering Group. *London MDC pilots*: Mush Ahmad, Data Manager; Donna Chung, Head of Centre for Cancer Outcomes, North Central and East London Cancer Alliance (formerly London Cancer, hosted by UCL Partners); Dr David Graham, Consultant Gastroenterologist; Dr Andrew Millar, Consultant Gastroenterologist; Sara Taiyari, Senior Project Manager; London MDC clinical teams. *Oxford MDC pilot*: Dr Claire Friedemann Smith, SCAN Researcher; Prof. Fergus Gleeson, Consultant Radiologist; Dr Shelley Hayles, Planned Care and Cancer Clinical Lead; Zoe Kaveney, Senior Project Manager; Dr Brian Nicholson, Macmillan GP and Senior Clinical Researcher; Oxford MDC clinical team.

## Supplementary information

Supplementary Information

## Data Availability

Pseudoanonymised data were supplied by the Multidisciplinary Diagnostic Centres (MDC)—ACE (Cancer Research UK) is the custodian of the data on behalf of the MDCs and for the duration of the analysis—the data are not publicly available at present.
