# Supplementary material for: First results from five multidisciplinary diagnostic centre (MDC) projects for non-specific but concerning symptoms, possibly indicative of cancer
Source: Br J Cancer. 2020 Jul 6;123(5):722–9. doi: 10.1038/s41416-020-0947-y (PMC7462853; doi:10.1038/s41416-020-0947-y)
Supplement: Supplementary file 1 — Supplementary Information [file 41416_2020_947_MOESM1_ESM.pdf]

Supplementary Information A (sensitivity analyses):

Table A1 and A2 shows the results of sensitivity analyses relating to missing data items within the study and assess the likely impact of the missing data by omitting sites where, for each variable considered in turn, no data were recorded for 20% or more of patients. The tables also show an alternate approach to addressing missing data items within the study and provides sensitivity analyses based on the calculation of a 'best case' and 'worst case' scenario for each variable. This is achieved by replacing all missing values with, in turn, the highest and lowest possible values of the variables.

Results:

Regressions conducted using the approaches outlined above did not provide strong evidence to warrant the reconsideration of conclusions reached in the study.

Table A1: previous 1° care consultations

**Previous 1° care consultations**	**Main results**	**Only projects with nearly complete data**	**Best case (assume missing is <=3)**	**Worst case (assume missing is >3)**
**Number of projects included**	5	2 (Airedale, Oxford)	5	5
**Number of patients in those projects**	2961	855	2961	2961
**Number (%) of patients with data item present**	1610 (54%)	839 (98%)	2961 (100%)	2961 (100%)
**Significance**	**0.002**	**0.031**	**0.002**	0.16
**Category**	Odds ratio (95% confidence interval)			
**<= 3**	1.00	1.00	1.00	1.00
**> 3**	**0.27 (0.12-0.62)**	**0.21 (0.05-0.87)**	**0.28 (0.12-0.63)**	0.83 (0.64-1.08)

Table A2: Duration of symptoms

**Duration of symptoms**	**Main results**	**Only projects with nearly complete data**	**Best case (assume missing is <1 week)**	**Worst case (assume missing is >6 months)**
**Number of projects included**	5	2 (Airedale, Oxford)	5	5
**Number of patients in those projects**	2961	855	2961	2961
**Number (%) of patients with data item present**	1949 (66%)	794 (93%)	2961 (100%)	2961 (100%)
**Significance**	**0.038**	*0.12*	**0.016**	**0.021**
**Category**	Odds ratio (95% confidence interval)			
**<1 week**	1.00	1.00	1.00	1.00
**1-4 weeks**	*1.62 (0.37-7.12)*	*1.40 (0.17-11.73)*	**1.83 (1.23-2.73)**	*1.62 (0.37-7.12)*
**5-12 weeks**	*1.09 (0.25-4.79)*	*0.86 (0.10-7.18)*	*1.24 (0.84-1.83)*	*1.09 (0.25-4.79)*
**3-6 months**	*1.08 (0.25-4.70)*	*1.20 (0.14-10.01)*	*1.22 (0.84-1.77)*	*1.08 (0.25-4.70)*
**>6 months**	*0.74 (0.17-3.29)*	*0.57 (0.07-4.92)*	*0.84 (0.54-1.30)*	*0.83 (0.19-3.59)*
